# Predictors of long- term survival after pelvic ring fractures in geriatric patients – five-year results of a prospective observational study of 134 patients

**DOI:** 10.1007/s00068-025-03030-2

**Published:** 2025-12-18

**Authors:** Julia Lenz, Jeanne Zwar, Ludwig Oberkircher, Martin Bäumlein, Vanessa Ketter, Juliana Hack, Daphne Eschbach, Ulf Bökeler, Steffen Ruchholtz, Tom Knauf

**Affiliations:** 1 Present Address: Marburg University, School of Medicine, Center for Orthopaedics and Trauma Surgery, University Hospital Gießen and Marburg GmbH, Baldingerstraße, 35043 Marburg, Germany; 2 Helios Hospital Schwerin, Wismarsche Straße 393-396, 19055 Schwerin, Germany; 3 Hospital of Friedrichshafen, Röntgenstraße 2, 88048 Friedrichshafen, Germany; 4 Helios Hospital Kassel, Bergmannstraße 32, 34121 Kassel, Germany; 5 Marienhospital Stuttgart, Böheimstraße 37, 70199 Stuttgart, Germany

**Keywords:** Pelvic ring fracture, FFP, 5-year mortality, Risk factors, Geriatric patient

## Abstract

**Purpose:**

The incidence of geriatric fractures, particularly pelvic fragility fractures, is rising due to demographic changes. While fractures located at the proximal femur remain the most common fracture in older adults, the number of pelvic fractures has constantly increased, especially following low-impact trauma such as falls. This study aims to evaluate the 5-year survival rate and identify factors influencing survival in geriatric patients with fragility fractures of the pelvis.

**Methods:**

A prospective single-center observational study included 134 patients aged 60 and older with fragility fractures of the pelvis from June 2012 to December 2016. Patients were contacted 5 years post-fracture to assess survival. Barthel Index, Instrumental Activities of Daily Living (IADL), comorbidity scores, living situation and fracture related data were collected. Statistical analyses were performed using IBM SPSS.

**Results:**

Of the 134 patients, 103 were re-examined after 5 years, revealing a mortality rate of 62.1%. The mean age of participants was 79.67 years, with a higher prevalence of females (82.5%). Survivors exhibited significantly better pre-fracture Barthel Index (91.79 vs. 82.35, *p* = 0.003) and IADL scores (6.38 vs. 4.11, *p* < 0.001). The ASA score and age-adjusted Charlson Comorbidity Score were also lower among survivors, indicating a correlation between pre-fracture functional status and long-term survival.

**Conclusion:**

Our Results indicate that survival outcomes for patients with fragility fractures of the pelvic are poor, highlighting an urgent need for tailored treatment protocols, which are currently lacking for pelvic fractures. Despite a survival rate of 37.9%, the treatment of these fragile patients remains warranted. Key predictors of survival include the pre-fracture Barthel Index and IADL scores, alongside lower ASA and Charlson Comorbidity Scores. We recommend assessing these factors upon admission to enhance mortality risk prediction and ensure more focused care for at-risk patients.

**Supplementary Information:**

The online version contains supplementary material available at 10.1007/s00068-025-03030-2.

## Introduction

Due to demographic changes, the incidence of geriatric fractures is still rising. Different epidemiological studies predict an age over 65 years in 25% of the world’s population by 2030 [1, 2]. While proximal femur fractures remain the most common fracture in the elderly, pelvic fragility fractures are becoming increasingly important in clinical practice as their incidence increases [1, 3, 4].

In contrast to high-energy traumata, which lead to pelvic fractures in younger patients [5], a low impact trauma, such as a simple fall, is the most frequent cause of fragility fracture in the elderly [6, 7]. Furthermore osteoporosis, with its high incidence especially in older females, leads to different radiological morphologies and stability ranges [8]. With these differences in the trauma mechanism and anatomical characteristics compered to younger patients, pelvic fractures in the geriatric population need other considerations regarding the most appropriate treatment [9].

While many investigations regarding long-term mortality, functional outcomes and quality of life in patients with typical geriatric fractures, such as proximal femur fractures, have been published in the past [10, 11], the evidence about these subjects in fragility fractures of the pelvis is still low. Although the German Federal Joint Committee (G-BA) has developed detailed treatment plans for patients with proximal femoral fractures, there are currently no specific treatment algorithms for those with pelvic ring fractures. Despite significant efforts to clarify treatment strategies for patients with fragility fractures of the pelvis such as multidisciplinary treatment concepts focusing on geriatric care, no mandatory algorithms have been developed [12].

Previous literature has already demonstrated the influence of factors such as age, sex, ASA score, Barthel Index, residential status and the Charlson comorbidity index on long-term survival in geriatric patients with proximal femoral fractures [13]. Therefore, we expected similar results when investigating the influence on geriatric pelvic ring fractures.

With a survival of only 38% within 5 years of follow- up [13], the long-term mortality of geriatric hip fractures remains high, although several multidisciplinary clinical pathways already exist, to improve life expectancy, functional outcomes and quality of life [12, 14]. It can be assumed that the long-term mortality in geriatric patients with pelvic fractures is comparably high. 

Therefore, the aim of this study was to evaluate the 5-year survival rate and to identify influencing factors for survival in patients with fragility fractures of the pelvis.

## Materials and methods

From June 2012 to December 2016, we included 134 patients in a prospective single- center observational study, who were admitted to our level-1-trauma center. In 2016, as part of the German Trauma Network, the trauma center treated 54 severely injured patients with an Injury Severity Score (ISS) of at least 16. Of these patients, 15% were over 70 years old [15].

All patients in the prospective observational study suffered from a fragility fracture of the pelvis and were 60 years or older. We excluded patients, who remained ambulatory, polytraumatized patients (injury severity score ≥ 16), patients with an isolated fracture of the acetabulum and patients with a malignancy-related fracture. As a part of our multidisciplinary treatment protocol for geriatric, many patients were discharged to a geriatric rehabilitation institution [16].

### Baseline data, treatment, influencing factors and survival

For the present analysis we contacted all patients 5 years after index fracture by phone. Each patient or their legal representative gave their written informed consent. A questionnaire for the following information was sent: housing, nursing care situation, Barthel index [17] and IADL [18] (instrumental activities of daily living). Data regarding the survival of the patients, we could not reach by phone, were collected at the local registration offices.

The following baseline data were documented from all participants at the beginning of hospital stay: age, gender, residential status, pre-fracture IADL [18] and Barthel Index [17] as well as at time of admission, ASA Score [19] and aged- adjusted Charlson Comorbidity Score [20]. During the index stay in hospital, the following data were collected: type of fracture (fractures were classified with the AO/OTA classification and FFP classification system [7]), fracture treatment (surgical vs. non-surgical), duration of hospital stay, Barthel Index and IADL.

We obtained the approval by the Ethics committee of the Philipps University of Marburg (AZ 10/20).

### Data management and statistical analyses

Data were collected in an Excel database (Microsoft Office 365, Microsoft Corporation Redmond, WA, USA). IBM SPSS statistics 25 (Statistical Package for the Social Science, IBM Corporation, Armonk, NY, USA) was used for statistical analysis. Data were presented as means, standard deviations and frequencies. For differences between both groups (survivors vs. non survivors) we used bivariate analysis. The normal distribution test was performed using the Shapiro-Wilk test. If no normal distribution was detected either the Mann-Whitney-U-Test or the Kruskal-Wallis-Test were performed. Nominal scaled variables were analysed using the Pearson-Chi-Quadrat-Test. Statistical significance was defined as *p* < 0.05.

We also carried out a binary logistic progression. The dependent variable has 2 values. Due to the multicolingiality and strong correlation of the different scores (CCI, ASA, Barthel and IADL), we only included the CCI in the model.

## Results

### Annual mortality

Of all 134 patients a total of 103 patients were re-examined 5 years after suffering the index fracture. 31 patients were lost to follow-up due to a lack of information about survival, which was collected by contacting the patients, their legal representatives, or local registration offices. We excluded them from the analysis completely.

**Fig. 1:**
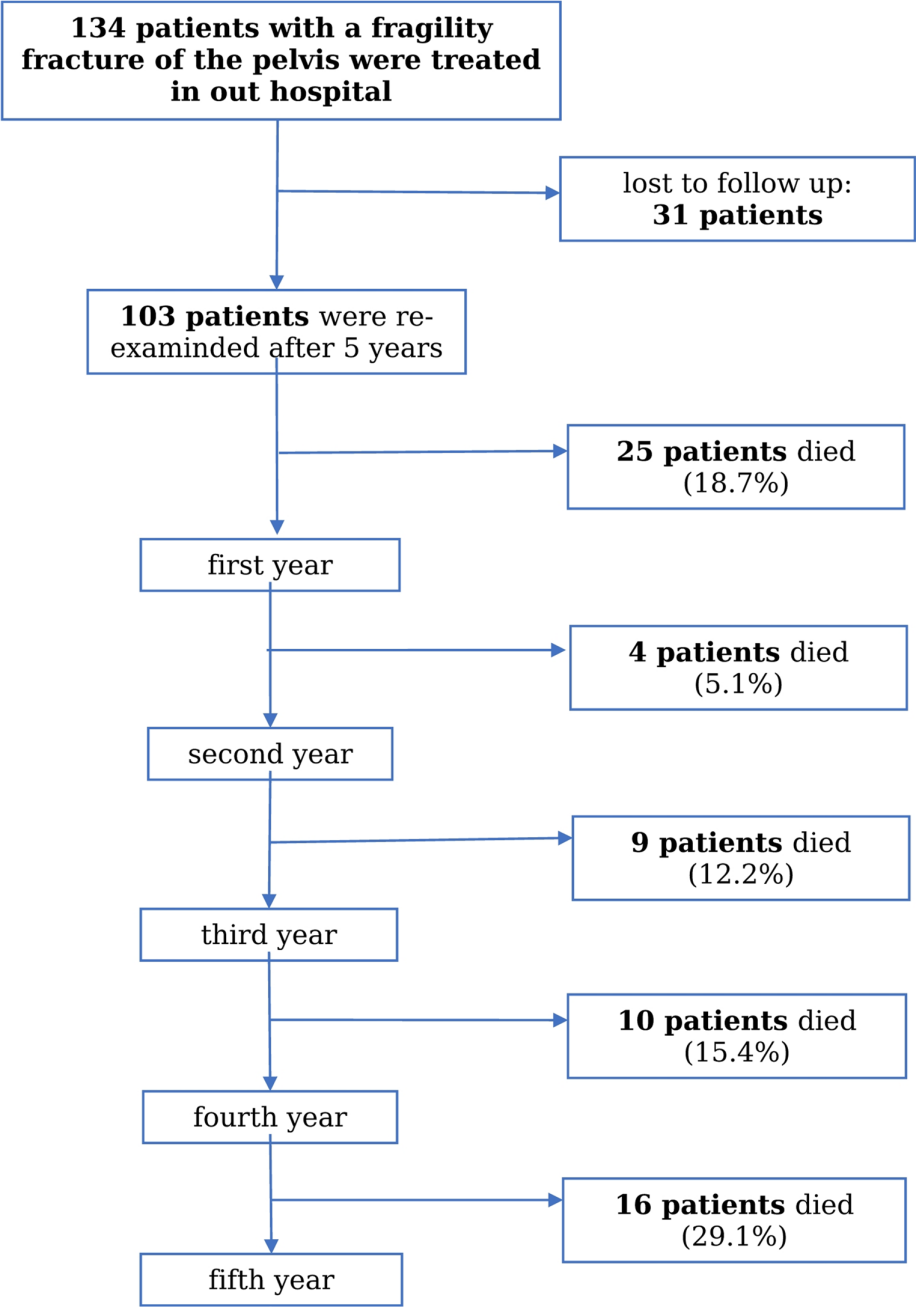
Annual mortality

**Fig. 2:**
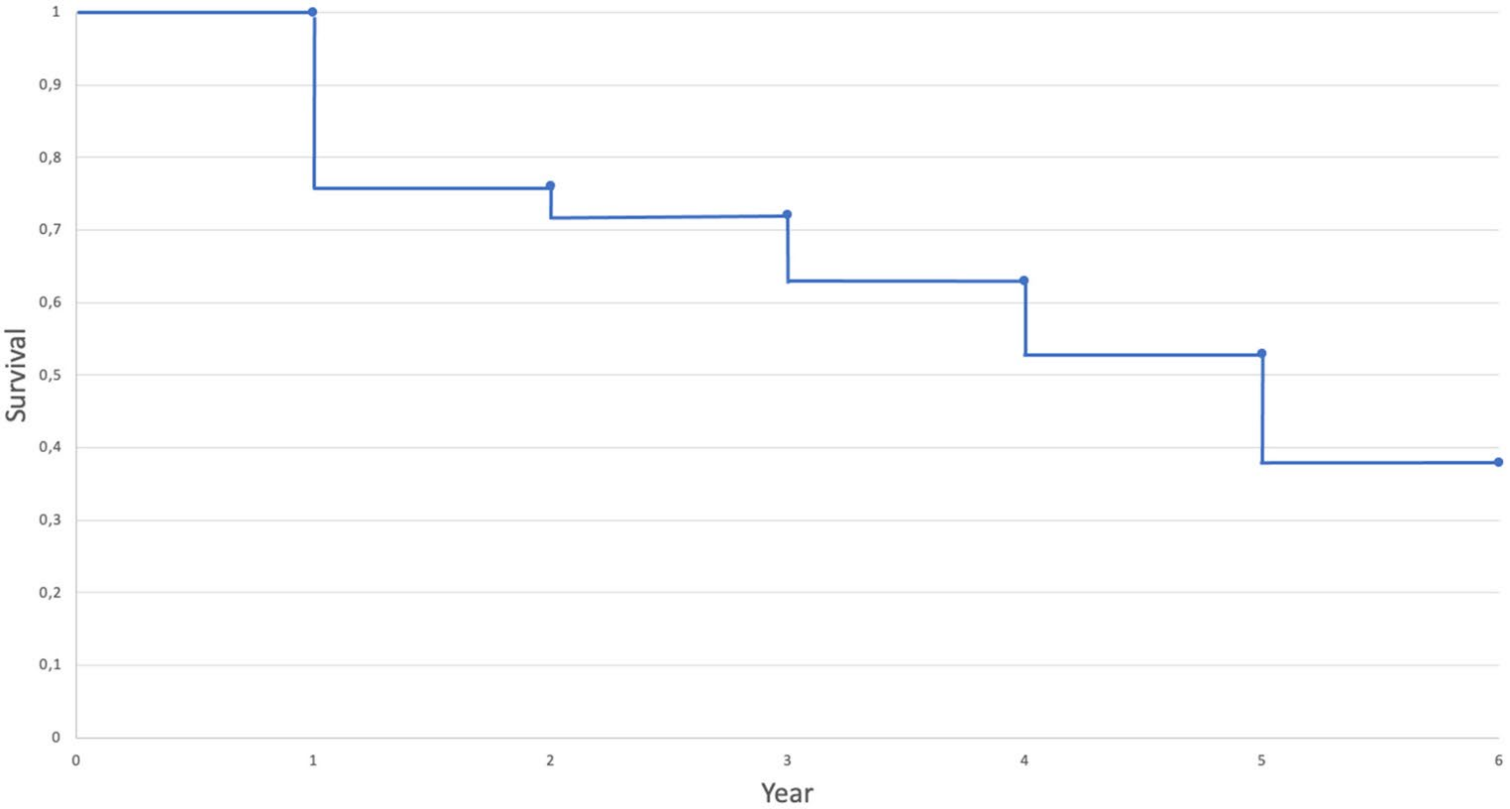
Kaplan Maier Curve showing the survival 6 years after fragility fracture of the pelvis

A detailed distribution regarding the mortality is shown the flow chart (Fig. 1). Of all re-examined patients 18.7% (*n* = 25) died within the first year, 5.1% (*n* = 4) within the second, 12.2% (*n* = 9) within the third, 15.4% (*n* = 10) within in the fourth and 29.1% (*n* = 16) within the fifth year after the index fracture. Due to that the mortality 5 years after fragility fracture of the pelvis was 62.1% (*n* = 64) (Fig. 2) .

### Baseline characteristics

The mean age of all patients was 79.67 years (SD 7.22, 95% CI 78.6–81.2). Compared to the deceased patients, the survivors were a little younger (78.1 vs. 80.63 years) without a significant difference (*p* = 0.086). The majority of our patients were female (*n* = 85; 82.5%). Regarding the gender distribution there was no significant difference between the deceased patients and the survivors (*p* = 0.526) (Table 1).

**Table 1:** Baseline characteristics at admission to hospital

Moment of admission to hospital	All (*n *= 103)	Deceased (*n *= 64; 62,1%)	Survivors (*n *= 39; 37.9%)	*p*-value
Age (years)				0.086
Mean	79.67 (SD 7.22)	80.63 (SD 7.16)	78.1 (SD 7,14)	
Range	61-95	61-94	64-91	
Gender (%)				0.526
Male	18 (17.5%)	10 (15.6%)	8 (20.5%)	
Female	85 (82.5%)	54 (84.4%)	31 (79.5%)	
Residential status				0.914
Nursing home	11 (10.7%)	7 (10.9%)	4 (10.3%)	
No nursing home	92 (89.3%)	57 (89.1%)	35 (89.7%)	
ASA Score				*p *< 0.001
1	1 (1.0%)	1 (1.6%)	0 (0%)	
2	33 (32.0%)	12 (18.8%)	21 (53.8%)	
3	61 (59.2%)	44 (68.8%)	17 (43.6%)	
4	5 (4.9%)	4 (6.3%)	1 (2.6%)	
5	3 (2.9%)	3 (4.7%)	0 (0%)	
Charlson comorbidity score	5.1 (SD 1.8)	5.7 (SD 1.8)	4.03 (SD 1.24)	0.001
Barthel index				0.003
Mean	85.92 (SD 18.44)	82.35 (SD 19.84)	91.79 (SD 14.26)	
Range	20-100	20-100	50-100	
IADL				*p *< 0.001
Mean	4.97 (SD 2.89)	4.11 (SD 2.78)	6.38 (SD 2.51)	
Range	0-8	0-8	0-8	

### Residential status

Most of the patients lived at home before admission to hospital (*n* = 92; 89.3%) and only 11 patients lived in a nursing home (10.7%). There was no significant difference between the deceased patients and the survivors (*p* = 0.914).

### ASA score

The majority of our patients had severe pre-existing illnesses. Due to that more than 80% of all patients were categorized as ASA 2 (*n* = 33; 32.0%) or ASA 3 (*n* = 61; 59.2%). The average ASA score of the deceased patients was 2.9 (95% CI 2,8 − 3,1) and of the survivors 2.5 (95% CI 2,3 − 2,7). The ASA score shows a significant association on the mortality (*p* < 0.001).

### Age-adjusted Charlson-Comorbidity-Score

The age-adjusted Charlson-Comorbidity-Score was significantly higher in the group of the deceased patients (*p* < 0.001). The average value of the survivors was 4.0 (95% CI 3.6–4.4) and of the deceased patients 5.7 (95% CI 5.3–6.2). Using the binary logistic progression model, CCI was also significant.

### Barthel and IADL

The long-term survivors had a significantly higher pre-fracture Barthel Index, 91.79 (SD 14.26; 95% CI 87.2–96.4) points vs. 82.35 (SD 19.84; 95% CI 77.4–87.3) points (*p* = 0.003), and a higher IADL, 6.38 (SD 2.51; 95% CI 5.6–7.2) points vs., 4.11 (SD 2.78; 95% CI 3.4–4.8) points (*p* < 0.001).

### Type of fracture

The most common fracture according to the FFP-classification by Rommens et al. was the FFP 2b fracture (*n* = 38; 36.9%). 30 patients had a FFP 1a fracture (29.1%), 14 patients a FFP 2c fracture (13.6%), 9 patients a FFP 2a fracture (8.7%), four patients a FFP 3a (3.9%) and 3b fracture (3.9%), two patients had a FFP 4b fracture (1.9%) and one patient a FFP 1b (1.0%) and 3c fracture (1.0%). The fracture type showed no significant association on the mortality of both groups (*p* = 0.528) (Table 2).

**Table 2:** Characteristics during hospital stay

Period in hospital	All (*n* = 103)	Deceased (*n *= 64; 62,1%)	Survivors (*n *= 39; 37.9%)	* p*-value
Fracture classification (FFP)				0,52
1a	30 (29.1%)	18 (28.1%)	12 (30.8%)	
1b	1 (1.0%)	0 (0%)	1 (2.6%)	
2a	9 (8.7%)	8 (12.5%)	1 (2.6%)	
2b	38 (36.9%)	22 (34.4%)	16 (41.0%)	
2c	14 (13.6%)	9 (14.1%)	5 (12.8%)	
3a	4 (3.9%)	2 (3.1%)	2 (5.1%)	
3b	4 (3.9%)	2 (3.1%)	2 (5.1%)	
3c	1 (1.0%)	1 (1.3%)	0 (0%)	
4a	0 (0%)	0 (0%)	0 (0%)	
4b	2 (1.9%)	2 (3.1%)	0 (0%)	
4c	0 (0%)	0 (0%)	0 (0%)	
Treatment				0,82
Surgical	41 (39.8%)	26 (40.6%)	15 (38.5%)	
Non-surgical	62 (60.2%)	38 (59.4%)	24 (61.5%)	

### Treatment

Most of the patients were treated non- operatively (*n* = 62; 60.2%). The non- surgical treatment included a full weight bearing mobilization, physiotherapy and analgesia. 41 patients underwent surgery (39.8%). The most common surgical procedure was a closed reduction of the posterior pelvic ring by a cemented sacroiliac screw (*n* = 32, 78.1%). The way of treatment (surgical vs. non-surgical) did not show a significant association on the mortality (*p* = 0.828) (Table 2).

## Discussion

The aim of this study was to investigate the 5-year survival after a fracture of the pelvic ring in geriatric patients. Furthermore, the study aimed to identify predictors of the long-term survival.

In our study population, the mortality rate was 62.1%. Survivors exhibited significantly better pre-fracture Barthel Index scores (91.79 vs. 82.35, *p* = 0.003) and Instrumental Activities of Daily Living (IADL) scores (6.38 vs. 4.11, *p* < 0.001). Furthermore, the ASA score and the age-adjusted Charlson comorbidity score were also lower among survivors.

In the following, the results of this investigation will be discussed in the context of the literature.

In summary, the analysis of our study group showed that only 37.9% of all patients were still alive 5 years after their pelvic ring fracture. In contrast to the expectation of the highest mortality within the first year after the fracture, the mortality varied over the survival period of 5 years (Fig. 1). Knauf et al. investigated the long-term survival after geriatric hip fractures [13]. They showed the highest mortality within the first year after hip fracture. This might be due to a postoperative weakened immune system, medication with accompanying symptoms and a lower degree of mobility. In contrast to this investigation the majority of our study patients deceased within the fifth year after fracture (29.1%). However, there is no evidence to explain this due to the multi-morbid character of geriatric patients and therefore a huge number of possibilities for the death. Schmitz et al. investigated the patient-related quality of life after pelvic ring fractures in geriatric patients [21]. They showed a two-year mortality rate of 29,3% without mentioning the exact number of deceased patients per year. Höch et al. analyzed the survival rate in geriatric patients with a lateral compression fracture of the pelvis and showed a 2-year mortality rate of 30% [22]. With a two-year mortality of 23.75% in our study population, this rate was slightly lower than that reported by Schmitz et al. and Höch et al. However, Ghassibi et al. showed a 1-year mortality of 23%, which is similar to our results [23]. Ghassibi and colleagues investigated the mortality rate and influencing factors after low-energy pelvic ring fractures in geriatric patients. With an overall survival rate of only 37.9% in our study group five years after fracture, this result is comparable to the rare previous published data for patients with geriatric pelvic ring fractures [24, 25] and similar to the survival rate after proximal femoral fractures reported in the literature [13, 26, 27]. Compared with the mortality rate in the general population, which is 4% for 80-year-old women and 6% for men of the same age [28], the annual mortality rate in our study population was much higher at 5–29%. Furthermore, these results suggest that the impact of a low-energy pelvic fracture may affect mortality at least within five years of injury. Therefore, further prospective studies focusing on pelvic fractures and involving a longer follow-up period could be useful.

Our study showed significant differences between the deceased patients and the survivors in several factors. The group of the survivors had a higher Barthel index at time of admission (91.8 vs. 82.3; *p* = 0.003), a higher IADL before trauma (6.4 vs. 41.1; *p* < 0.001), a lower ASA score (2.5 vs. 2.9; *p* < 0.001) and a lower age- adjusted Charlson Comorbidity Score (4.0 vs. 5.7; *p* < 0.001). Leung et al. evaluated the prognosis of acute pelvic fractures in elderly patients [29]. In contrast to our results they found no significant increase in the 2-years mortality due to preexisting medical conditions. Breuil et al. examined the outcome of osteoporotic pelvic fractures in 60 patients with a mean follow up of 29 months [30]. In 7 patients data regarding the cause of death were available. Death was predominantly related to cardiovascular events, which could be related to pre-existing conditions. Comparable to our results Ghassibi et al. showed a significant influence of the number of comorbidities on mortality after low-energy pelvic ring fractures in the elderly [23]. Desmet et al. identified a high number of comorbidities as an independent predictor of one-year mortality as well [31]. They investigated predictors of mortality one year after pelvic fractures in 282 geriatric patients. Comparable results regarding the influence of a high ASA score and Charlson Comorbidity Score have already been published for geriatric patients with proximal femoral fractures [13, 32, 33]. Patients, who were still alive 5 years after their fracture, showed a higher IADL and Barthel index at time of admission. Desmet et al. showed comparable results. Although they did not analyze the Barthel index and IADL, they showed a significant relation of living in a nursing home pre-fracture and using of a walking aid to one year mortality following a pelvic ring fracture. People, who are living in a nursing home, are often more multimorbid and correspondingly have a lower Barthel index and IADL than a comparable group of people living in their own home [34]. Furthermore using a walking aid is one of the items of the Barthel index [17].

Our study showed no differences between the deceased patients and the survivors in age, gender, fracture classification and therapy (surgical vs. non-surgical). While multiple authors showed the male sex as an factor, which influences the long term survival after proximal femoral fractures [35], there exists evidence for geriatric patients with pelvic ring fractures as well. Desmet et al. identified the male gender as an independent predictor of one-year mortality. While age was marginally non-significant (*p* = 0.086), Desmet et al. and Ghassibi et al. showed a significant influence of the patients age on the one-year survival after pelvic ring fracture [23, 31]. As an interesting finding there was no significant differences between both groups (survivors vs. deceased patients) regarding the therapy (surgical vs. non-surgical). Höch et al. showed a significant higher 2-year survival in patients with lateral compression fracture, who underwent surgery [22]. Osterhoff et al. compared early operative treatment with non-operative treatment of fragility fractures of the pelvis in a propensity-matched pair multicenter study with 230 patients. In this study, non-operative treatment was linked to improved survival rates in the first two years. However, those patients who underwent early operative treatment and survived the initial two years demonstrated better long-term survival outcomes [36]. Moreover, our study showed no significant difference between both groups regarding fracture classification. This finding is comparable to the results of Desmet et al., who did not find a difference between deceased patients and survivors in fracture classification [31]. Omichi et al. investigated 552 patients with fragility fracture of the pelvis and did not find a significant difference in survival rates between fracture types as well [37]. However, they only included patients, who were treated non-surgical.

Nevertheless, the data was collected several years ago. Bearing this in mind, it is worth asking whether patients with fragility fractures of the pelvis are still treated in the same way. According to the Fragility Fracture of the Pelvis (FFP) classification system, treatment depends on this classification [7, 38]. However, there is still a lack of prospective studies. Stable fractures are usually treated conservatively, whereas surgery is recommended for unstable fractures and those involving the posterior ring [7, 38]. However, the superiority of operative treatment for stable fractures involving the posterior ring remains unproven [39]. In cases of unstable fractures (FFP III/IV), the literature recommends not only stabilizing the posterior ring, but also addressing the anterior pelvic ring [38]. There are different minimally invasive treatment options for the anterior pelvic ring, which could affect survival and functional outcomes in patients with more unstable pelvic fractures [40]. Surgical treatment at our university hospital remains stage-appropriate and has not changed significantly since the study was conducted.

Due to the recent optimization of interdisciplinary care within the framework of orthogeriatric co-management for geriatric patients, we anticipate a positive impact on outcomes and mortality, similar to those observed for patients with hip fractures [41].

## Limitations and strengths

This study has several limitations. First of all the study was conducted at a single center, which may limit the number of participants. Although 134 patients were included, the sample size may still be relatively small, especially when stratifying for specific variables, which could affect the statistical power of the analyses. Furthermore, some factors such as Barthel Index before trauma had to be collected retrospectively. Another limitation is the high number of patients, who were lost to follow up (*n* = 31). In addition, the study group consisted of geriatric patients. Mortality may therefore be related to the multimorbidity of these patients and not to the fracture itself. This is a fact that we have to take into account. Furthermore, the data was collected several years ago. The prospective study design, the examination of multiple factors influencing survival and the long period of the follow-up (5 years) are to be mentioned as strengths of this study. Moreover, to our knowledge it is the first study, which analyzes the 5-year mortality of geriatric pelvic ring fractures.

## Conclusion

This study examined the 5-year survival rate of geriatric patients with fragility fractures of the pelvis and identified factors influencing survival. The findings confirm poor results for those fragile patients, which are comparable to those of patients with proximal femoral fractures. Although the incidence of pelvic ring fractures in the elderly is increasing, there are still no specific treatment algorithms, unlike patients with proximal femoral fractures, for whom detailed treatment plans have been developed by the German Federal Joint Committee (G-BA). Nevertheless, with 37.9% of all patients surviving, the effort required to treat these fragile patients is still justified. Key predictors of survival included pre-fracture Barthel Index and IADL scores, both of which reflect the functional ability of patients. Additionally, a lower ASA score and Charlson Comorbidity Score were linked to improved survival. We therefore suggest evaluating the Barthel score, the Charlson comorbidity score and the IADL upon admission to better predict mortality rates and identify patients at risk. This could optimize outcomes for these patients in an orthogeriatric setting.

## Electronic supplementary material

Below is the link to the electronic supplementary material.

Supplementary Material 1

## Data Availability

The data that support the findings of this study are not openly available due to reasons of sensitivity and are available from the corresponding author upon reasonable request. Data are located in controlled access data storage at University Hospital Gießen and Marburg.
