# Supplementary material for: Predictors of long- term survival after pelvic ring fractures in geriatric patients – five-year results of a prospective observational study of 134 patients
Source: Eur J Trauma Emerg Surg. 2025 Dec 18;51(1):364. doi: 10.1007/s00068-025-03030-2 (PMC12715046; doi:10.1007/s00068-025-03030-2)
Supplement: Supplementary file 1 — Supplementary Material 1 [file 68_2025_3030_MOESM1_ESM.docx]

**Towards Wound segmentation from 3D Body Scans**

**Developing methods for military trauma surgeons**

# **Abstract**

Background: Modern wound and surgical care often uses 3D body scans as the input, and automated machine learning assessment of these artefacts offer increased efficiency and quality of care especially for military traumas.

Method: While segmentation of two-dimensional images is a highly common deep learning task, there is less previous work on 3D segmentation. This paper chronicles our efforts to segment gunshot wounds from 3D scans of patients injured during armed conflict. Working on the outputs of 3D imaging software, we investigated various machine learning methods including 3D-UNet and conventional segmentation techniques. The method presented here is a novel color and material-based segmentation approach that utilizes 2D projection for 3D objects.

Results: Preliminary experiments demonstrate a segmentation accuracy of 95%, with a Dice coefficient of 45% (±29% standard deviation), measured based on correctly identified vertices. It is important to note that the dataset was manually annotated by the authors, which may influence the evaluation metrics. Additionally, a web-based user interface has been developed to enable interactive exploration of the results, serving as a prototype for future integration into full-scale surgical planning software.

Conclusion : Creating accurate assessment tools for war wounds can help save precious times in contexts where doctors are already under a lot of pressure. This article presents segmentation methods as a first step towards assessment of 3D wounds.

Keywords: 3D Segmentations, Automated Wound Assessment, Gunshot Wounds, Machine Learning, Wound Analysis, Projection Methods, Trauma Surgery

# **Introduction**

Armed conflicts incur Gunshot wounds (GSWs) representing a significant concern in both forensic science and trauma medicine, necessitating precise and efficient methods for their assessment and analysis. The advent of three-dimensional (3D) imaging technologies has revolutionized the way medical professionals and forensic experts visualize and interpret complex injuries, providing a more comprehensive understanding of the spatial relationships and characteristics of these wounds (Kumar et al., 2020). Segmentation of war wounds from 3D body scans is a critical step in this process, enabling accurate measurement, analysis, and documentation of the injuries sustained. Most of the previous works regarding the segmentation of wounds focuses on 2D images segmentation. The developed segmentation models can reach accuracies up to 90% (Wang et al., 2020) [1].

This study investigates the feasibility of automating the segmentation of gunshot wounds from three-dimensional (3D) scans acquired in a military surgical context. Working with a limited set of high-resolution scans obtained under wartime conditions, our goal is to develop an end-to-end computational pipeline capable of supporting both forensic analysis and clinical decision-making. Specifically, this work addresses three main challenges: identifying suitable algorithms for segmenting complex 3D wound geometries; evaluating the achievable accuracy of these methods when applied to real-world, unlabelled trauma data; and designing an interactive web-based interface to visualize and assess the segmentation outputs in a clinically relevant manner.

2 Theory

### *2.1 3D Body Scanning*

Each scan is stored in the .obj format, a widely used and compatible 3D file format. The dataset includes the following components for each scan:

- **Geometric data**: Vertices and faces that define the 3D shape of the body and the wound. (.obj)
- **Texture files (RGB images)**: Mapped onto the geometry to provide realistic skin and tissue coloration, including visible bleeding or bruising. (.png, .jpg…)
- **Material files (.mtl)**: Describe surface properties such as glossiness, reflectivity, and transparency, enhancing the realism of the model.

These 3D assets can be easily visualized using tools like Blender. Blender, allows interactive inspection of the scans, where users can rotate, zoom, and examine the wound areas under various lighting conditions. This capability is crucial for manual validation, expert review, and better understanding of wound morphology.


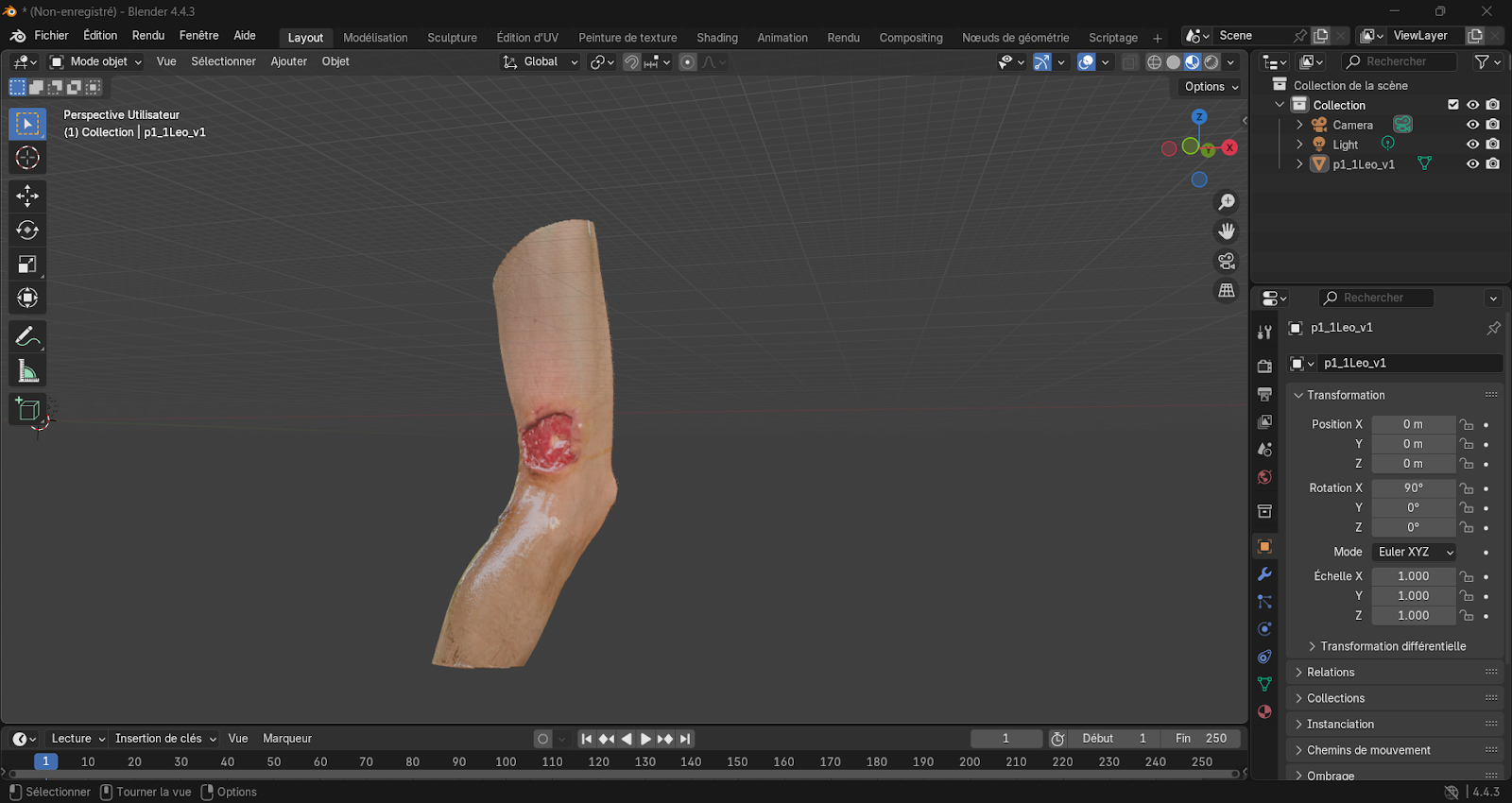


Fig 1 3D Gunshot Wound Scan Visualization in Blender

The scans composing our dataset, have been sent to us by the Kharkiv regional hospital in Ukraine using LEO and SPIDER scanners produced by Artec3D. These scanners reach accuracies up to 0.1 mm. Our dataset is composed of N different scans, of N2 different wounds.

### *3D Segmentation*

Segmentation in 3D imaging refers to the process of identifying and isolating specific regions of interest within a 3D model. This step is essential for accurate analysis, measurement, and documentation of injuries, whether for medical or forensic purposes. Segmenting a wound allows professionals to focus computational and clinical attention on the affected area, facilitating diagnosis, and treatment planning.

Techniques such as thresholding, region-based segmentation, and edge detection have been traditionally used; however, machine learning and deep learning approaches are becoming increasingly prevalent. In particular, convolutional neural networks (CNNs) have demonstrated strong performance in automating wound segmentation tasks by enabling more precise delineation of complex wound boundaries (Ronneberger et al., 2015)[2]. While this study does not propose a new CNN architecture, we leverage an existing model DeepSkin to evaluate the feasibility of applying CNN-based segmentation to 2D projections of 3D wound data. The segmentation process typically involves preprocessing steps to enhance image quality, such as noise reduction, contrast adjustment and other. Following preprocessing, the segmentation algorithm is applied to identify and classify different regions. The segmentation process can also include postprocessing steps to enhance the algorithm results depending on segmented data or type of performed segmentation.

Moreover, the integration of 3D visualization techniques can provide clinicians with a comprehensive view of the wound, facilitating better decision-making and patient management. By leveraging 3D models generated from segmented data, healthcare professionals can assess wound healing over time, monitor changes in size and shape, and tailor treatment approaches accordingly. Segmenting GSWs from unlabelled 3-D files has proven difficult due to some intertwined obstacles. The most pressing issue is the lack of labelled data. Many segmentation methods, particularly those based on supervised machine learning, use annotated examples to learn the characteristics and properties that distinguish wounds from healthy tissue and body parts. Without these annotations, such methods are rendered ineffective. Unsupervised methods, which do not require labels, struggled to generalize across the dataset’s diverse and irregular wound patterns.

### *Military traumas*

One of the most prevalent types of injuries in military settings is penetrating trauma, particularly from firearms. Gunshot wounds can cause extensive damage to soft tissues, bones, and vital organs, often resulting in significant blood loss and requiring urgent surgical intervention (Hoffman et al., 2018)[3]. The management of such injuries typically involves debridement, stabilization of fractures, and reconstruction of damaged tissues, which may include the use of grafts or prosthetics. Blast injuries are another common type of trauma seen in military patients, often resulting from improvised explosive devices (IEDs) or other explosive ordnance. These injuries can lead to a combination of penetrating wounds, blunt force trauma, and secondary injuries caused by flying debris (Elder et al., 2015)[4]. The complexity of blast injuries often necessitates a multidisciplinary approach to care, addressing not only the physical injuries but also potential psychological impacts, such as post-traumatic stress disorder (PTSD). Shrapnel injuries, which occur when fragments from explosives penetrate the body, can result in both soft tissue and skeletal injuries. These injuries are particularly challenging due to the unpredictable nature of the damage caused by high-velocity projectiles. Surgical management often involves meticulous removal of foreign bodies and reconstruction of affected areas (Huang et al., 2016)[5].

Moreover, military trauma can lead to unique complications, such as infection and delayed healing, particularly in cases involving extensive soft tissue loss or bone exposure. The use of advanced wound care techniques, including negative pressure wound therapy and bioengineered skin substitutes, has shown promise in improving healing outcomes for complex military wounds (Miller et al., 2017)[6].

### *Evaluating Segmentation Accuracy*

Benchmarking image segmentations is essential for evaluating the performance of various segmentation algorithms, particularly in medical imaging and other applications where precision is critical. Several statistical metrics are commonly employed to assess the accuracy and effectiveness of segmentation results. Here are some of the key statistics used in the evaluation of image segmentations:

**Dice Similarity Coefficient (DSC):** The Dice coefficient is one of the most widely used metrics for comparing the similarity between two sets of data, typically the ground truth and the segmented output. It is calculated as:

$DSC=\frac{2\mid A\cap B\mid}{\mid A\mid+\mid B\mid}$

where (A) is the set of pixels in the ground truth, and (B) is the set of pixels in the segmentation. (Dice, 1945)[7].

**Sensitivity (Recall):** Sensitivity measures the proportion of actual positive cases (true positives) that are correctly identified by the segmentation algorithm. It is calculated as:

$Sensitivity=\frac{TP}{TP + FN}$

where (TP) is the number of true positives and (FN) is the number of false negatives. This metric is particularly important in medical imaging, where missing a positive case can have significant consequences (Powers, 2020)[8].

**Specificity:** Specificity assesses the proportion of actual negative cases (true negatives) that are correctly identified. It is calculated as:

$Specificity = \frac{TN}{TN + FP}$

where (TN) is the number of true negatives and (FP) is the number of false positives. This metric helps evaluate the algorithm's ability to correctly identify non-target regions (Powers, 2020)[8].

**Precision:** Precision quantifies the accuracy of the positive predictions made by the segmentation algorithm. It is defined as:

$Precision = \frac{TP}{TP + FP}$

A high precision indicates that the algorithm has a low rate of false positives, which is crucial in applications where false alarms can lead to unnecessary interventions (Powers, 2020)[8].

### *Clustering Methods*

Clustering is an unsupervised learning technique used to group data points based on similarity. It is widely employed in image analysis tasks, including segmentation of medical images, where it helps identify regions with similar characteristics such as color, intensity, or texture (Jain et al., 1999)[9]. K-means is a popular partitioning method that divides data into *k* clusters by minimizing intra-cluster variance. In image processing, particularly in color image segmentation, k-means can be used to separate regions based on pixel intensities or color values. While it is computationally efficient and easy to implement, it assumes spherical clusters and is sensitive to initial conditions, making it less effective in handling complex structures or noisy data (Pham et al., 2000)[10].

Web API

Web Application Programming Interfaces are standardized interfaces that allow remote systems to communicate over the internet using protocols such as HTTP. In the context of medical image analysis, Web APIs provide an efficient way to access advanced processing tools, models, or datasets hosted on remote servers or cloud platforms (Schreiber et al., 2020)[11]. These APIs facilitate:

- **Remote image processing**, such as segmentation, classification, and feature extraction.
- **Scalability**, by offloading computation to high-performance backends.
- **Interoperability**, enabling integration across various programming environments

# **2. Methods**

This retrospective experimental study was conducted between March and August 2025. It involved anonymized 3D scan data collected from a military hospital in Kharkiv, Ukraine. Ethical approval was obtained from the World Medical Association (WMA). Due to the retrospective and de-identified nature of the data, informed consent was waived.

## *2.1 Dataset Description*

The 3D data used in this project originates from high-resolution scans acquired at a hospital in Kharkiv, Ukraine. These scans were taken from living soldiers suffering traumas in armed combat, with a focus on gunshot. The scanning process was performed using a high-resolution 3D scanner, which is capable of capturing fine anatomical details of the human body, including wound structures and subtle tissue deformation. There was a total of 38 scans, of the following limb: arms 3; elbow 2; legs 8; hands 5; fingers 2; unidentified 18

## *2.2 The Projections Method*

The method we decided to use relies on 2D projections of the 3D object at different angles. These projections can be segmented using Deepskin (Deepskin .2024)[12], a neural network (with U-Net architecture) for wound segmentation. Then we can project back the 2D segmentation back on the 3D object.

#### Preprocessing

The first step to generate a projection was loading a 3D mesh using open3d. The wavefront files used image files for texturing. Using a “UV map” each triangle of the mesh can be mapped to a pixel, representing its color. A custom function was implemented to process the vertex colors of the mesh accordingly.

##
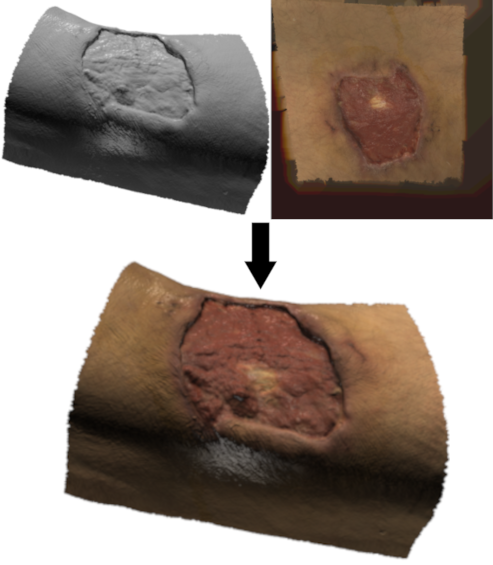


Fig 2: an untextured mesh, a texture file, and a textured mesh(using vertex colors).

#### 2D Projections

To begin making a projection we first decided we would place the camera on the +Z axis:

- Rotate the mesh accordingly to the desired angle
- Create a mask of the mesh vertex excluding the ones with normals pointing outwards the camera. This was achieved by using the dot product of the normal and the positive z axis [0, 0, 1] , thereafter checking whether the resulting value is positive (meaning the pixel should be visible) or not.
- Create a cubic grid of voxels, according to the desired resolution.
- Iterate through all the triangles of the mesh
- Iterate through all the voxels that the triangle at least touches on the X and Y axis, and select the voxels on the middle eight of the triangle. (This means only one layer of voxel is processed for each triangle. This approximation saves a bit of processing time and is not considered acceptable due to high resolutions)
- For all the voxels, if their center falls inside the triangle and if they are the front most processed voxels, mark the X and Y coordinate in the image corresponding to the same X and Y for the voxel with the mean of the color of the 3 vertices that compose the triangle. To keep track of which voxel is the front most that has been processed we use a depth map and update it with the Z coordinate each time a new voxel is selected for image coloration.

To do the 26 first projections we used this process and defined the 26 angles evenly placed around the object. We normalized these angles and in the case of a Gimbal Lock (Luhta, 2010)[13] the method was designed to set the yaw to 0.


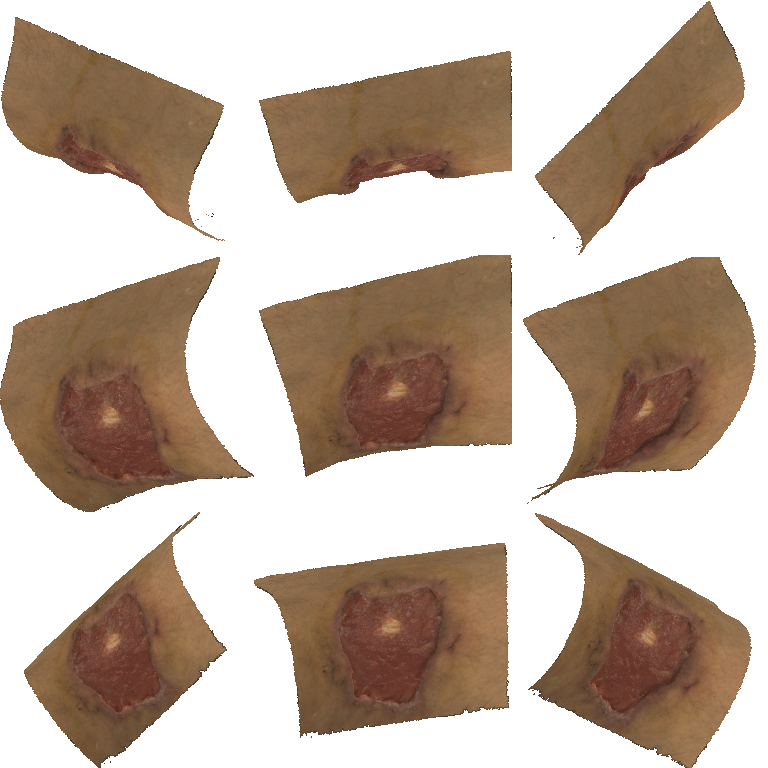


Fig 3 The 9 front projections of a wound

#### First Segmentations

Once the 26 first projections were made we used Deep Skin on each generated image to get an estimation of the best angle for looking at the wound. On the projections where the camera is looking at the back of the object, the segmented image will be mostly white meaning the CNN won't find any wound. On the other more pertinent angles, we might find the wound. We have empirically selected the best angle by compute a weighted average of each angle of projection with its weight being : 4 * the PWAT(Photographic Wound Assessment Tool PWAT – Revised 2010)[14] of the wound at the angle * the ratio of non-background pixels in the image + the PWAT * the non-background ratio of the 4 +- 45° pitch, +- 45° yaw segmentations around the selected angle. To get the corresponding 4 projections we had to normalize angles and set the yaw to 0 in case of Gimbal Lock. The formula includes the neighbour projections as a factor for the final weight to avoid selecting an angle where a side projection would provide a bad angle.

##
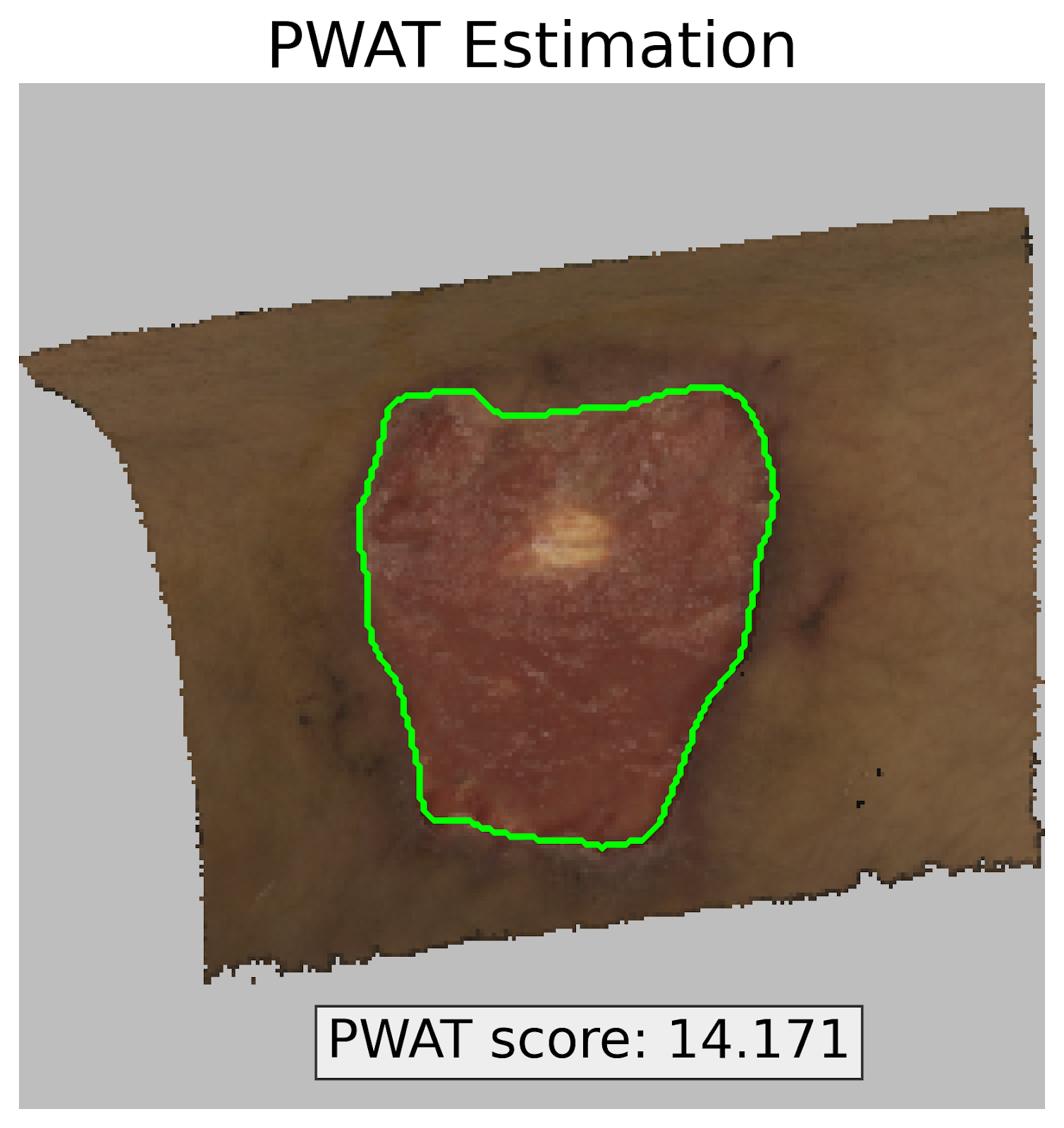

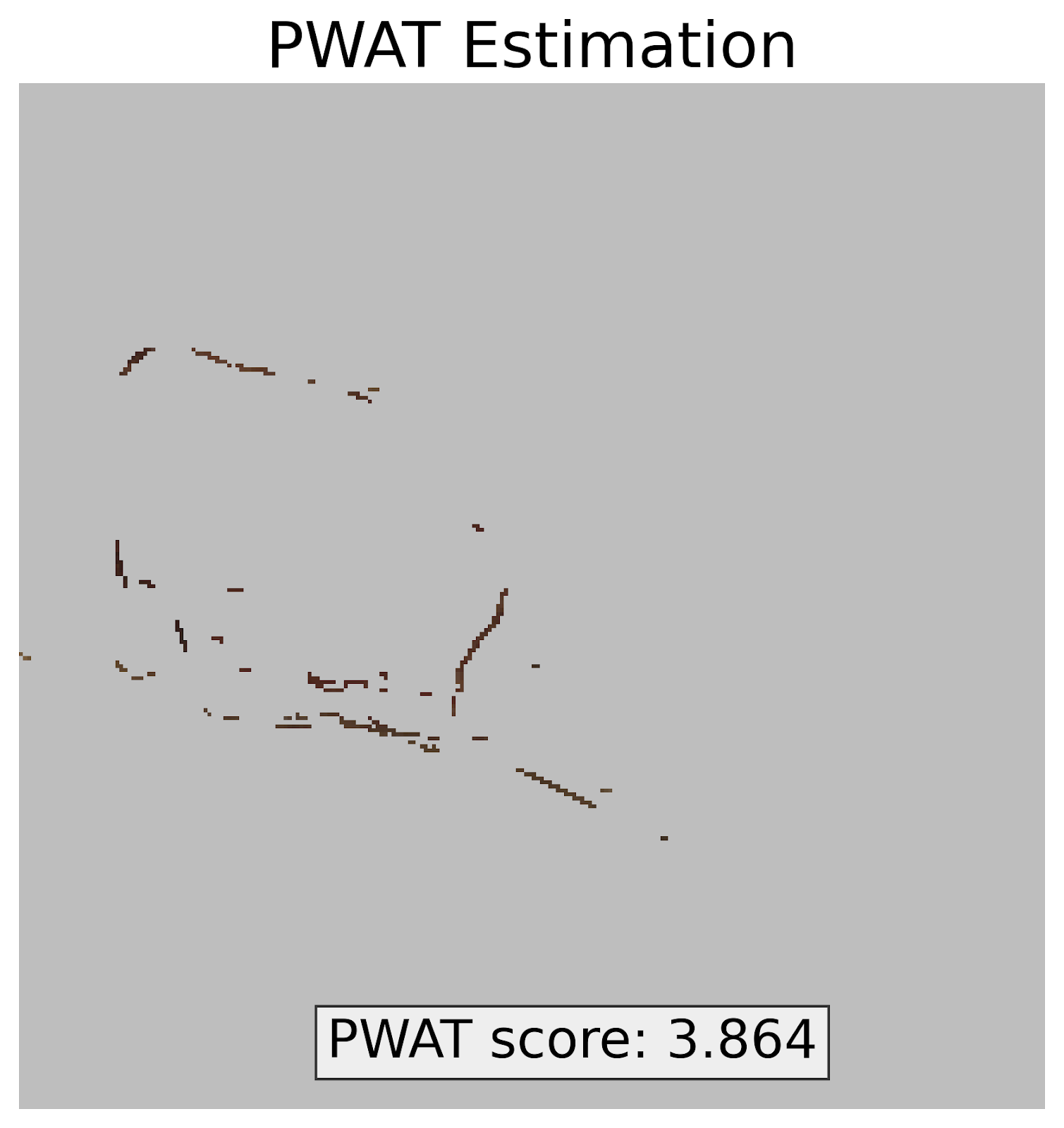


Fig 4 Deepskin’s PWAT estimation on the bottom-front and back projections


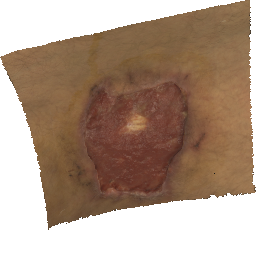


Fig 5 Estimated best angle for the final projections

#### Second Segmentations

Once we had retrieved this final angle estimation we computed new projections. As previously stated, 1 at the said angle and 4 at the 4 +- 45° pitch, +- 45° yaw angles. When doing so we computed the voxel-vertices map for the final step.

#### Transfering the 2D segmentations to the mesh

We are now at the final step of the process. It simply consists of marking all the vertices of the mesh that are stored inside of the projection and that fall inside the wound masks. To do this we use the mask we have computed earlier and the voxel to vertices map. We can also display the result by changing the vertex colors to red.


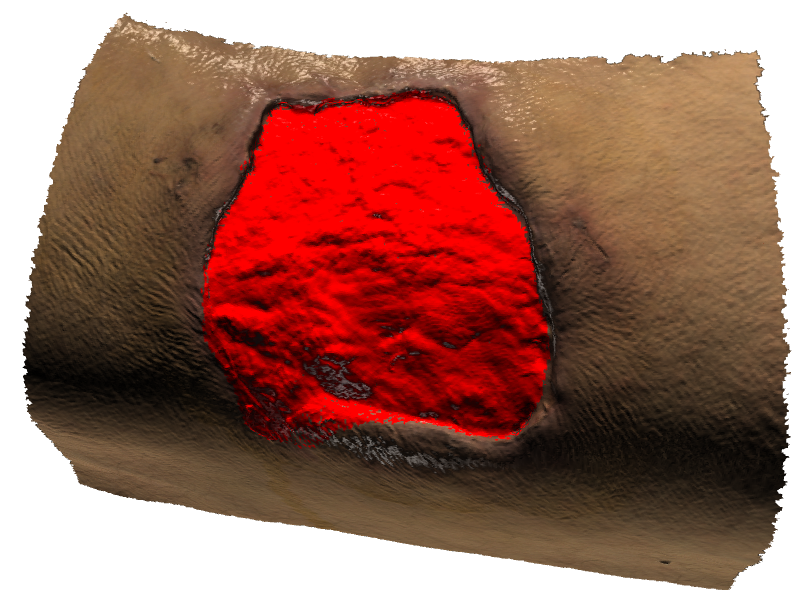


Fig 6 Segmentation result on the mesh

### *2.3 Validation method*

To test our results we use 4 standard metrics regarding segmentation : accuracy, sensitivity, specificity and the Dice score. We calculate in terms of the number of vertices that have been correctly segmented.

### 2.4 Feasibility

The execution time can be quite heavy, especially with detailed mesh, yet with lower resolution one, with 50 000 triangles used for the demo, the method can be as fast as 5 minutes to run. This was measured on a laptop with Ubuntu, 16Go of RAM and a 10th gen I7 processor.

### 2.5 Web Demo API

To facilitate the evaluation of our various segmentation methods, we developed a web-based API designed to streamline the testing process. This system enables users to either upload their own 3D scan files (.obj, .mtl, .png) or select from a curated set of preloaded models available within the interface. Once a model is selected, the user can choose the segmentation technique to be applied to the wound. The platform then generates an interactive 3D visualization displaying both the original (unsegmented) model and the resulting segmentation, allowing for clear comparative assessment.

The backend of the application was developed in Python, while the frontend was implemented using standard web technologies: HTML, CSS, and JavaScript. We employed Uvicorn as the ASGI server to ensure efficient communication between the backend and frontend components. For the segmentation tasks, we integrated the DeepSkin model, which performs 2D wound segmentation on projected images and provides an estimate of the PWAT (Percentage of Wound Area to Total)—a useful metric for assessing wound severity. This architecture supports a flexible and modular environment for testing, visualization, and iterative refinement of segmentation approaches.

### **3. Results**

*3.1 Segmentation*

To be able to evaluate results for the segmentation we had to create masks for the wounds. To do so we used blender’s painting tool and painted the wounds in black to generate new texture files.


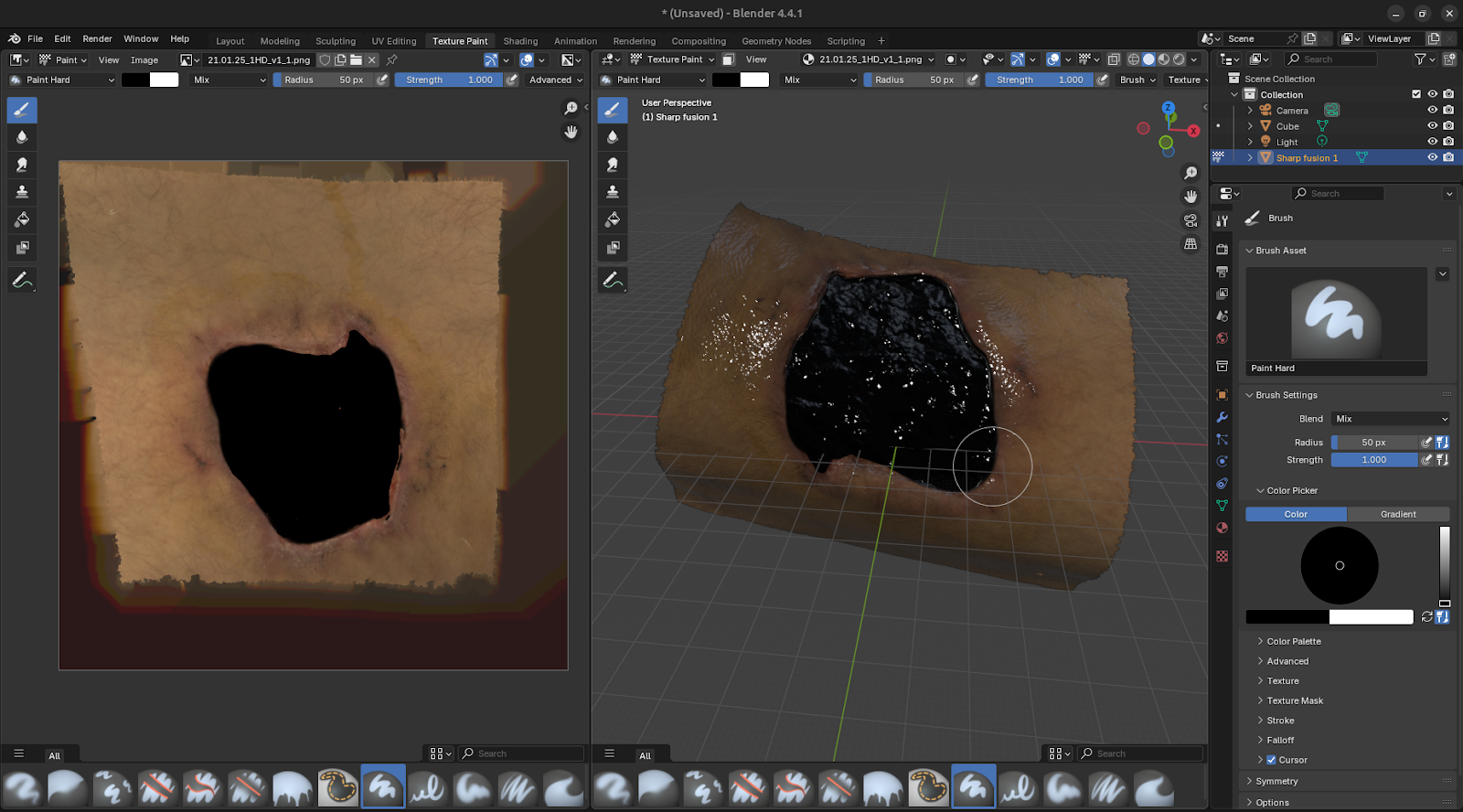


Fig 7 Blender painting of the wound

The final results are evaluated based on the number of vertices we assigned correctly. We measure this using 4 usual metrics when it comes to segmentation : Accuracy, sensitivity, specificity and the Dice score. Table 1 presents the results.

Table 1. Mean (SD) of the measured benchmarks.

| **Model** | **Accuracy** | **average sensitivity** | **average specificity** | **average dice** |
| --- | --- | --- | --- | --- |
| Projections method  (38 wounds tested) | 94.67% (6.03) | 52.83% (32.40) | 97.65% (4.12) | 55.48% (29.14) |

# The results show that we have a good accuracy and specificity but for a good part of the wounds we have some issues with sensitivity, meaning we do find the wound but not entirely. 3 wounds also were not detected at all, which we might consider an error. (We should include the figure that got removed)

# *3.2 User Interface*


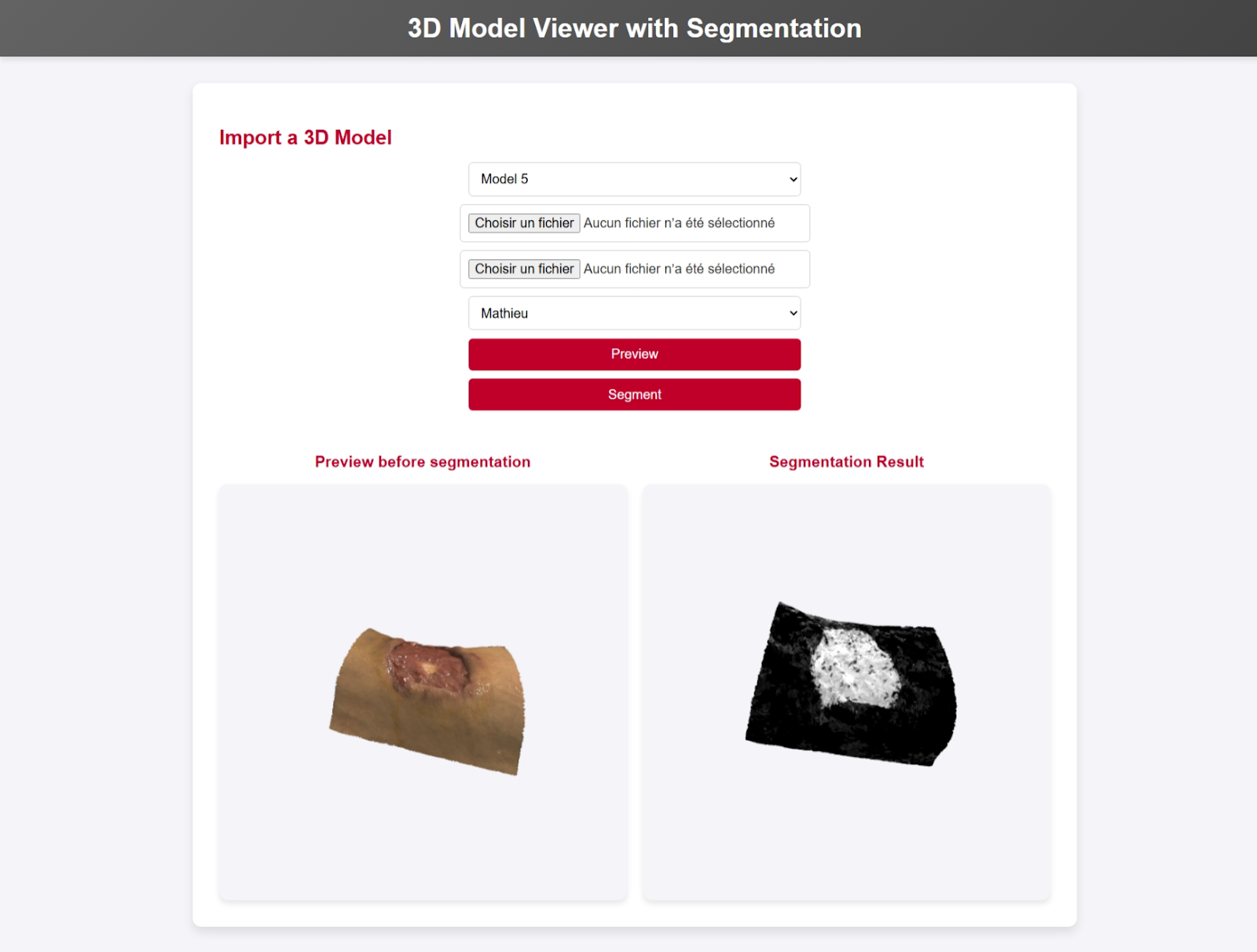


Fig 8 Web Interface

The user interacts with a web interface designed for the visualization and segmentation of textured 3D models. This interface, built using Three.js and connected to a FastAPI backend, enables the dynamic loading of .obj models along with their associated texture files (.png) and material files (.mtl). The user can either select a preloaded 3D model available in the application or upload their own files. Once the model is loaded, it can be previewed directly in the 3D scene before any processing takes place.

A dropdown menu allows the user to select a segmentation method. When a method is chosen, an API call is triggered to perform the segmentation on the server side. Once the processing is complete, the model is reloaded with a new segmented texture, which is rendered in the 3D viewer. The interface also provides the ability to toggle between the original texture and the segmented result, making visual comparison straightforward.

### **4. Discussion**

We initially began with the projection method, then we tried CNN, then we tried clustering, inspired by its common application in medical image segmentation tasks like tumor detection in MRI scans. Our hypothesis was that a machine learning model could learn to identify wound boundaries by being trained on the geometric and texture data contained in the .obj files. This method turned out to be unfeasible in a short time because our dataset lacks labels. To train the model effectively in supervised learning, it requires annotated examples, specifically regions that are explicitly marked as wounds. The algorithm can’t perform in the absence of these labels because it was unable to differentiate wounds from the surrounding tissues. Unsupervised learning methods, which group data according to innate patterns without the need for annotations, were also taken into consideration. However, in contrast to their success with more standardized datasets like 2D segmentation, where tissue boundaries are frequently more predictable, these methods had difficulty generalizing across the irregular and highly variable wound shapes in our scans. This early failure made clear how crucial labelled data is to machine learning, which led us to look for other approaches.

One limitation of this study lies in the heterogeneity of the wound appearances across the dataset. Some cases presented ambiguous or ill-defined wound boundaries, which posed challenges even for human reviewers. This variability may have impacted the reliability of the annotations and, consequently, the evaluation of the segmentation algorithms. Future work could benefit from the construction of a high-quality reference dataset composed exclusively of clearly delineated wounds to improve both training and validation consistency.

Existing 3D imaging software, such as 3d Slicer or Blender, present another limitation. Although these platforms include tools such as region growing and edge detection, they require significant manual effort to refine segmentations, making them unsuitable for large-scale or urgent applications. Despite having some great results on some wounds, this method presents some flows. Our main issue is the sensitivity of our model, which also brings down our mean dice score. To address such issues we might try to implement new solutions like filling small holes inside our mask, or extending the mask based on the nearby vertex colors. 2D models present way more reliable results, with mean sensitivities and mean dice scores around 90 % (Wang et al., 2020). As a potential avenue for future optimization, we consider the possibility of decimating the 3D meshes to reduce computational load, which may in some cases facilitate the segmentation process. One hypothesis is that decimating the mesh only during the initial segmentation phase could offer a useful trade-off between processing time and segmentation accuracy, though this remains to be tested experimentally.

**Conclusions**

The study has shown that 3D segmentation can be achieved via a method of projections to multiple 2D planes. While results are less consistent than the state of the art 2D methods, the technique serves as an important first stage in the automated assessment of gunshot wound severity. A web API was also created to demonstrate the method and raise interest for machine learning in surgery. Future plans include refinement of the methods, better annotation and gamified demonstrations.

**Funding**

The project was funded by the Mainz Foundation for Science.

**Clinical trial number: not applicable**

# **Ethics**

No ethics permission has been obtained as data was gathered in a nation under wartime conditions being invaded by a foreign aggressor and under a state of emergency. The military surgeons at the clinics have guaranteed that data were anonymised without any risk of identification. They provided the images with permission to use in research as part of our collaboration with the aim of saving human lives.

# **References**

[1] Wang C, Anisuzzaman DM, Williamson V et al. Fully automatic wound segmentation with deep convolutional neural networks. Sci Rep. 2020;10:21897. doi:10.1038/s41598-020-78799-w

[2] Ronneberger O, Fischer P, Becker A. U-Net: Convolutional networks for biomedical image segmentation. In: MICCAI 2015. Springer; 2015. p. 234-41.

[3] Hoffman MK, O’Connor JH, McCarthy ML. Gunshot wounds: A review of the literature. J Trauma Nurs. 2018;25(3):179-84. doi:10.1097/JTN.0000000000000352

[4] Elder JH, McGowan JE, Kahn S. Blast injuries: A review of the literature. J Trauma Acute Care Surg. 2015;78(2):377-85. doi:10.1097/TA.0000000000000497

[5] Huang J, Wang Y, Zhang Y. Management of shrapnel injuries: A review. Injury. 2016;47(6):1158-65. doi:10.1016/j.injury.2016.02.004

[6] Miller AC, Kauffman JM, Mendez A. Advanced wound care for military trauma: A review. J Spec Oper Med. 2017;17(3):64-70

[7] Dice LR. Measures of the amount of ecological association between species. Ecology. 1945;26(3):297–302

[8] Powers DMW. Evaluation: From precision, recall... J Mach Learn Technol. 2020;2(1):37–63

[9] Jain AK, Murty MN, Flynn PJ. Data clustering: a review. ACM Comput Surv. 1999;31(3):264–323. doi:10.1145/331499.331504

[10] Pham DL, Xu C, Prince JL. Current methods in medical image segmentation. Annu Rev Biomed Eng. 2000;2:315–37. doi:10.1146/annurev.bioeng.2.1.315

[11] Schreiber A, Fellmann M, Vössing M et al. Web-based medical image processing. Methods Inf Med. 2020;59(S 01):e46–e56. doi:10.1055/s-0040-1701229

[12] Deepskin [Internet]. DeepSkin: Deep learning segmentation of chronic wounds. GitHub repository. 2024 [cited 2025 Aug 8]. Available from:<https://github.com/Nico-Curti/Deepskin>

[13] Luhta E. Gimbal Lock. In: How to Cheat in Maya 2010. Elsevier; 2010. p. 127–41. doi:10.1016/b978-0-240-81188-8.50006-0

[14] Photographic Wound Assessment Tool PWAT – Revised [Internet]. 2010 [cited 2025 Aug 8]. Available from:<https://www.southwesthealthline.ca/healthlibrary_docs/b.9.3b.pwatinstruc.pdf>

**Statements & Declarations**

This work was supported by internal research funding provided by Mainz Foundation for Science as part of a technological innovation initiative in battlefield medical imaging. No external funding or grant number is associated with this study.

The authors declare that they have no competing interests related to the content of this article.

As all data used in this study were fully anonymized and collected retrospectively, the requirement for informed consent and publication consent was waived under institutional protocol.
